# Expression and Localization of microRNAs in Perinatal Rat Pancreas: Role of miR-21 in Regulation of Cholesterol Metabolism

**DOI:** 10.1371/journal.pone.0025997

**Published:** 2011-10-11

**Authors:** Louise Larsen, Maiken W. Rosenstierne, Louise W. Gaarn, Annika Bagge, Lykke Pedersen, Christina M. Dahmcke, Jens H. Nielsen, Louise T. Dalgaard

**Affiliations:** 1 Department of Biomedical Sciences, University of Copenhagen, Copenhagen, Denmark; 2 Department of Science, Roskilde University, Roskilde, Denmark; 3 Niels Bohr Institute, University of Copenhagen, Copenhagen, Denmark; Cincinnati Children's Hospital Medical Center, United States of America

## Abstract

**Objective:**

To investigate the expression of pancreatic microRNAs (miRNAs) during the period of perinatal beta-cell expansion and maturation in rats, determine the localization of these miRNAs and perform a pathway analysis with predicted target mRNAs expressed in perinatal pancreas.

**Research Design and Methods:**

RNA was extracted from whole pancreas at embryonic day 20 (E20), on the day of birth (P0) and two days after birth (P2) and hybridized to miRNA microarrays. Differentially expressed miRNAs were verified by northern blotting and their pancreatic localization determined by *in situ* hybridization. Pathway analysis was done using regulated sets of mRNAs predicted as targets of the miRNAs. Possible target genes were tested using reporter-gene analysis in INS-1E cells.

**Results:**

Nine miRNAs were differentially expressed perinatally, seven were confirmed to be regulated at the level of the mature miRNA. The localization studies showed endocrine localization of six of these miRNAs (miR-21, -23a, -29a, -125b-5p, -376b-3p and -451), and all were expressed in exocrine cells at one time point at least. Pathways involving metabolic processes, terpenoid and sterol metabolism were selectively affected by concomitant regulation by miRNAs and mRNAs, and *Srebf1* was validated as a target of miR-21.

**Conclusions:**

The findings suggest that miRNAs are involved in the functional maturation of pancreatic exocrine and endocrine tissue following birth. Pathway analysis of target genes identify changes in sterol metabolism around birth as being selectively affected by differential miRNA expression during this period.

## Introduction

MicroRNAs (miRNAs) are small, single-stranded non-coding RNA molecules involved in post-transcriptional control of gene-expression of a wide number of genes. MiRNAs align and bind especially to 3′UTR sequences of their target genes and initiate either mRNA degradation or translational repression, resulting in reduced protein levels [1]–[3].

MiRNAs have been found to regulate many animal developmental events such as proliferation, differentiation and apoptosis [4]. Development of pancreas and islets of Langerhans is highly dependent on developmental timing controlling specification, neogenesis, proliferation and differentiation of individual cell types [5]. Removal of endogenous miRNAs at different embryonic time-points using *Dicer^(flox/flox)^* mice illustrate that miRNAs indeed are involved in the fetal development of pancreas, most notably the beta-cell lineage [6].

Numerous miRNAs have been reported to have roles in pancreatic beta-cells: MiR-124a targeting *Foxa2* [7], [8] and miR-9 controlling insulin exocytosis via its target *Onecut-2* [9]. MiR-375 is one of few miRNAs (along with miR-7) expressed mainly in adult islets and only marginally elsewhere [10]–[13], and controls a cluster of genes regulating cellular growth and proliferation, evident from studies of *miR-375^(−/−)^* mice, which are hyperglycemic and have decreased beta-cell mass [11]. Thus, miRNAs have important functions in mature beta-cells and for fetal development of beta-cells.

A burst of beta-cell replication and maturation takes place in the perinatal period [14]–[16]. The mechanisms regulating perinatal gene expression have not been described in details; and the miRNA profile of late developmental events in the pancreas has not been determined. The present study investigates the expression patterns of pancreatic miRNAs during the period of perinatal beta-cell expansion and maturation to identify sets of differentially regulated miRNAs. Subsequently, we determine the anatomical localization of these miRNAs. Additionally, the current miRNA expression profile and a corresponding mRNA expression profile from the same samples was used to investigate possible downstream target pathways of the differentially regulated miRNAs.

## Materials and Methods

### Ethics statement

All studies were conducted in accordance with institutional guidelines and approved by the Danish Animal Experiments Inspectorate. Permit ID: 2008-561-1515.

### Tissue-samples

Female Wistar rats, 10–11 weeks, were time-mated at Taconic, Denmark and transferred to local facilities one week prior to experiments. Animals had free access to food and water and were kept on a 12 hr light–12 hr dark cycle. The rats were killed at gestational day 20 (E20), immediately after birth (P0) or two days after birth (P2), and the offspring were decapitated. Pancreata were excised and placed in cold TRI Reagent (Sigma-Aldrich, St. Louis, MO) for RNA extraction. This was repeated in three independent experiments. Separately, protein lysates from pancreata were prepared using RIPA-buffer with a protease inhibitor cocktail and Tissue-LyserII (Qiagen, Copenhagen, Denmark).

### RNA extraction, qualification and sample preparation

Total RNA was extracted according to manufacturer's recommendations. RNA quality was measured using 2100 Bioanalyzer (Agilent Technologies, Santa Clara, CA, USA). Samples with a 28S/18S RNA ratio >2 and RNA integrity number >7 were used for arrays. Three biologically different RNA pools were generated for each time point by combining an equal amount of RNA from 3–5 offspring from each dam. A common reference pool was generated by combining the three biologically different RNA pools from all three time points.

### MicroRNA array analysis

RNA (1 µg) from each of the three biologically different, pooled samples and common reference were labeled with Hy3 and Hy5 respectively using miRCURY LNA Labeling Kit (Exiqon, Vedbaek, Denmark). Internal control synthetic Spike-in probes (Exiqon) were added to each labeling reaction. Labeling swaps were performed for technical duplicates of each sample. Labeled RNA was hybridized to miRCURY LNA microRNA Arrays ver.8.1 (Exiqon) in a HS400-Pro hybridization station (Tecan, Grödig, Austria). This array comprises probes for miRNA described in the miRBase 8.1 release of the miRNA registry (http://www.mirbase.org/) (462 human, 340 murine and 234 rat miRNAs).

### Data processing and analysis

Microarrays were scanned on an ArrayWoRx CCD-based scanner (Applied Precision, Issaquah, WA, USA), and analyzed using ImaGene and GeneSight software (Biodiscovery, Los Angeles, CA, USA). Data were normalized within arrays using intensity-dependent global normalization (LOWESS). The ratio between sample and reference was calculated in addition to the difference between the log_2_ transformed sample and reference. Quality of the data was assessed by plotting normalized signals of Hy3 against Hy5 and evaluating Spike-in controls. Slides showing linear labeling were included in the statistical analysis. Hence, sample 3 of P0 was rejected. Data with signal intensities below 25 were discarded. Statistical analysis was performed using the TM4∶MeV software package [17] and differentially expressed miRNA were identified using one-way ANOVA (cut-off p<0.01). Only miRNAs with greater than 1.5-fold increase or decrease in expression at any time point were used for further analysis. Hierarchical gene-tree clustering analysis was performed by TM4∶MeV using Euclidean distance and average linkage. MIAME compliant miRNA array data have been deposited in Array Express under the accession number E-MTAB-594.

*Northern blotting:* Total perinatal or INS-1E cell RNA (5 µg) was resolved in 15% TBE-Urea gels (Invitrogen, Novato, CA, USA), photographed and blotted to positive nylon membranes (Qbiogene, Montréal, Canada). Complementary locked nucleic acid (LNA) probes (Exiqon) for mature miRNAs were end-labeled with [γ-^32^P]dATP (Perkin Elmer, Waltham, MA, USA). Hybridization was performed using the ULTRAhyb-Oligo protocol (Ambion, Foster City, CA, USA).

### *In situ* hybridization (ISH)

Pancreata excised at E20, P0 or P2 were formalin-fixed and paraffin-embedded. For each time-point 3–4 different pancreata and 2–3 sections per miRNA were hybridized and studied. Sections were denatured 10 min at 42°C in 2.5 mU/ml Proteinase K (Roche, Hvidovre, Denmark), incubated 5 min in 4% paraformaldehyde, acetylated 10 min in 0.1 M triethanolamine pH 8.0/0.25% acetic anhydride and prehybridized for 1 hr at 22–25°C below probe Tm in hybridization mix (50% formamide/5x SSC/0.5 mg/ml yeast tRNA/1x Denhardt's solution supplemented with 9.2 mM citric acid if hybridization temperature was above 55°C). Complementary LNA probes (Exiqon) were DIG-labeled using DIG Oligonucleotide Tailing Kit 2^nd^ Generation (Roche) according to manufacturer's recommendations. 2.5 pmol DIG-labeled probes were added to hybridization mix, heated to 90°C then iced, applied to each section and hybridized overnight at 22–25°C below probe Tm. Sections were washed at 12–15°C below probe Tm in decreasing SSC concentrations and incubated 15 min at 37°C in 20 µg/ml RNase A. For immunostaining sections were incubated 2 hr in 1∶100 anti-Digoxigenin-AP (Roche), Vulcan Fast Red was applied and nuclei were counterstained with Haematoxylin Carazzi. Sections were mounted from xylene and imaged on a Leica DM 4000 B using Leica Application Suite software. Negative controls included sections without probe and with scrambled control probe (Exiqon) that bear no homology to any known miRNA sequence.

### *In silico* functional analysis

MiRanda (Microcosm at mirbase.org) [18] was used to predict mRNA targets of differentially regulated miRNAs. This search retrieved 728–994 predicted mRNA targets for each of the differentially regulated miRNAs. MessengerRNAs expressed in perinatal rat pancreas and regulated more than 2-fold from E20 to P2 were extracted. This dataset was based on Affymetrix array hybridization of the same samples as those used for miRNA array hybridizations (L.W. Gaarn and J.H. Nielsen, unpublished data). Comparison of miRanda predicted mRNA targets and differentially expressed Affymetrix gene-sets was performed using Venn diagrams [19]. Biological pathways were identified using Gene Ontology [20] and KEGG [21] terms and GeneCodis 2.0 software [22]. Default settings were used for GeneCodis 2.0 with p-values adjusted with the method of Hochberg and Benjamini [23].

### Cell culture and nucleofection

INS-1E cells (gift from Claes Wollheim, Geneva, Switzerland) were cultured as described previously [24]. 4·10^6^ cells were nucleofected with miR-21 and/or miR-29a LNA knock-down, or scrambled LNA oligonucleotide (Exiqon) using an Amaxa nucleofector (Lonza, Copenhagen, Denmark). Transfection efficiency was approximately 70%. Cells were harvested for RNA and protein 48 hr after transfection using TRI Reagent, or cells were trypsinized, counted and extracted for cholesterol.

*Real-time RT-Q-PCR:* Complementary DNA was synthesized from 1 µg RNA using iScript (BioRad, Copenhagen, Denmark) following manufacturer's recommendations. Q-PCR was performed using SYBR Green with melting curve detection on a Roche LightCycler instrument. For INS-1E *TfIIB* and for perinatal pancreas *Rpl13alpha* was used for normalization (described in [25], L.W. Gaarn and J.H. Nielsen, unpublished data). MiR-21 Q-PCR was performed on stem-loop primed cDNA according to [26] with *TfIIB* reverse primer present in the gene-specific cDNA synthesis. Oligonucleotide sequences used are listed in Table S3.

### Western blotting

Proteins (20 µg) were separated on 12% acrylamide gels, blotted to PVDF-membranes and Ponceau stained. After blocking membranes were incubated with antibodies for SREBF1 (Santa Cruz, sc-8984), SQLE (Santa Cruz, sc-49754), MnSOD (Stressgen, sod-111) or beta-actin (Abcam, A6276). As loading controls MnSOD was used on perinatal blots and beta-actin was used for cell culture extracts. HRP-conjugated secondary antibodies (DAKO, Glostrup, Denmark) and enhanced chemiluminescence (Pierce, VWR, Herlev, Denmark) were used for visualization.

### Reporter gene analysis

Oligonucleotides with predicted miRNA binding sites or 2 base mutated binding sites (in the seed sequence) were annealed, phosphorylated and cloned into pGL4.13 (Promega, Nacka, Sweden) in the 3′UTR of the *luc2* gene (using FseI/XbaI). Constructs with perfect complementarity to miR-21 (‘Perfect’) and this sequence scrambled (‘Scr’) were made as controls. All sequences were checked for introduction of other miRNA binding sites, and if this occurred in mutant constructs, other bases were changed to avoid this (Table S3). Resulting clones were sequenced, purified and used for transfection using Lipofectamine 2000 (Invitrogen) of INS-1E cells. Reporter constructs were cotransfected with pRL (Promega) to correct for differences in transfection efficiencies. Endogenous miR-21 was inhibited with 25 pmol/well antisense LNA-21 (Exiqon) cotransfected with reporter vectors (scrambled LNA oligonucleotides served as negative control). Twenty-four hours later cells were lysed and luciferase activities measured using the Dual-luciferase assay (Promega). To facilitate comparisons of individual experiments the mean of the pGL4.13 vector was adjusted to the same level in all 4 experiments and the data pooled. The resulting means are in arbitrary values.

### Cholesterol determination

Cells were extracted using chloroform∶isopropanol∶NP-40 (7∶11∶0,1) and total cholesterol determined using the Cholesterol Quantitation Kit according to instructions from manufacturer (MBL International, Nordic Biosite, Copenhagen, Denmark).

### Statistics

ANOVA was used to test for differentially expressed miRNAs, using a significance level of 0.01. The significance level for FDR adjusted p-values when using Gene-Codis was 0.05. In other experiments t-test was used to compare treatments, with a significance level of 0.05.

## Results

### Identification of miRNAs in perinatal rat pancreas on embryonic day 20, postnatal day 0 and postnatal day 2

This study investigated miRNAs expressed in the perinatal rat pancreas on embryonic day 20 (E20), postnatal days 0 and 2 (P0 and P2). The LNA-oligonucleotide array identified the presence of 108 known rat miRNAs (for complete list and heat-map see Fig.S1). A number of miRNAs are expressed at high intensities throughout the perinatal period, but are not regulated from E20 to P2 (listed with decreasing expression level: miR-298, -494, -292-5p, -503, -290, -320, let-7c, -327, -185, let-7b and let-7a).

**Figure 1:**
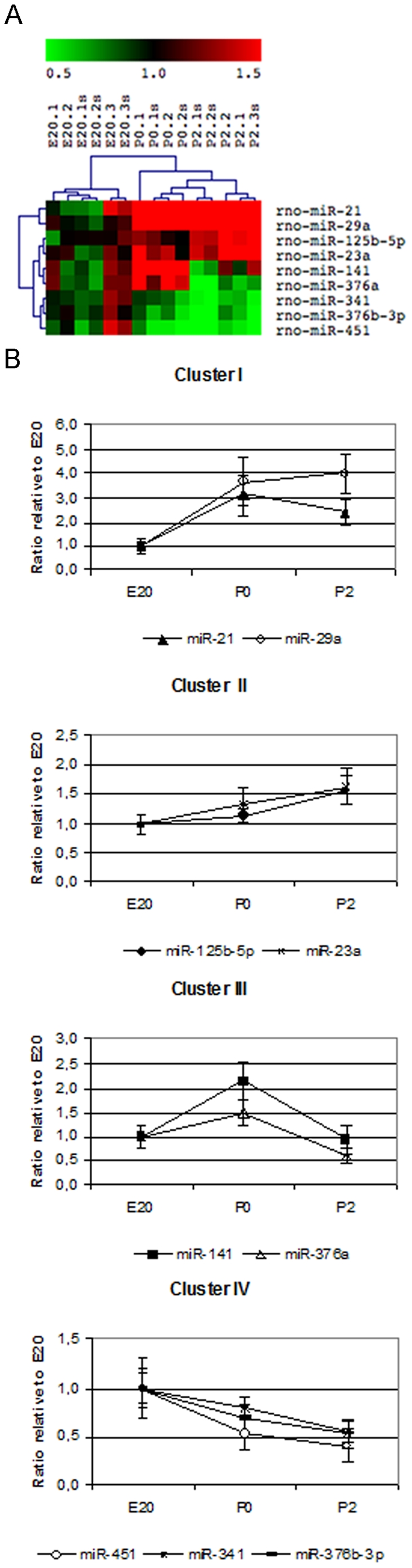
Microarray analysis of differentially expressed miRNAs in the perinatal rat pancreas. **A**. Heat-map showing hierarchical gene-tree and sample clusters of the 9 miRNAs that were significantly (p<0.01) regulated more than 1.5-fold. Sample to reference ratio for the different biological samples are shown. (Complete heat-map is shown in Fig.S1). **B**. Expression pattern of the miRNAs based on the gene-tree clustering. Shown is the ratio compared to E20 of the mean of sample to reference ratio with SD.

We identified 9 miRNAs that were differentially expressed and showed a more than 1.5-fold increase or decrease in expression level across the different time points from E20 to P2. These were: miR-21, -23a, -29a, -125b-5p, -141, -376a, -376b-3p, -341 and -451 (Fig. 1A). The intensities are in the middle range except for miR-341, which is expressed at a low level (data not shown).

To group miRNA with similar expression profiles, gene-tree clustering analysis was performed (Fig. 1B), which identified 4 specific expression patterns. Cluster I and II consisted of miR-29a and miR-21, and miR-125b-5p and miR-23a, respectively, and showed an increased expression at P0 and P2 compared to E20. Cluster III included miR-141 and miR-376a and had increased expression at P0 and then a decreased expression at P2 compared to E20. Cluster IV included miR-451, -341 and -376b-3p and their expression decreased from E20 to P2.

### Validation of microarray hybridizations using northern blotting

**Figure 2:**
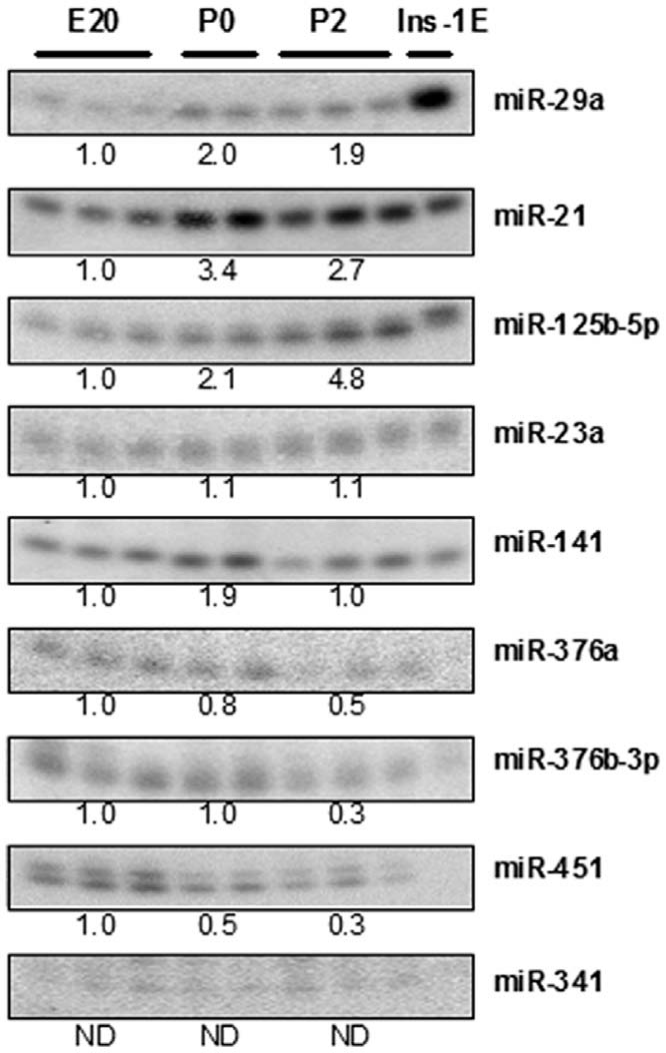
Validation of miRNA expression using northern blotting. Northern blots of the mature miRNAs are shown. Total RNA from INS-1E cells were used as a control for miRNA expression in beta-cells. Quantification of the mature miRNA bands is shown below each gel as the ratio of signal intensities compared to E20. (Images of whole gels are shown in Fig.S2).

We used northern blot analysis to verify the array results and to visualize the presence of pri-, pre- and mature miRNA (Fig. 2), which revealed mature miRNA for all the analyzed miRNA except miR-341. Because only pre-miR-341 was detectable by northern blotting, it was eliminated from further analysis (for images of whole blots see Fig.S2). Quantification of the mature miRNA bands for the remaining miRNAs showed a similar expression pattern as the array data and hence verified the array results. The exeptions were miR-376a, which show a similar expression pattern as miR-376b-3p; and miR-23a, whose levels did not significantly rise from E20 to P2 (Table S1). We detected two mature species for miR-451, which may be due to its unusual structure where the mature miRNA extends into the loop sequence [27]. INS-1E cells were used as a control for miRNA expression in beta-cells and all miRNAs could be detected in INS-1E cells except miR-376b-3p, -376a and -451. Interestingly, INS-1E cells contained precursor miRNA species for miR-451 and miR-376b-3p, but absence of mature miRNAs (Fig.S2). Thus, 3 out of the 9 miRNAs differentially regulated in our data-set show difference between regulation at precursor and mature miRNA levels either in pancreas or in INS-1E cells, suggesting cell type specific or temporal regulation of miRNA processing.

### Localization of differentially expressed miRNAs in perinatal rat pancreas

**Figure 3:**
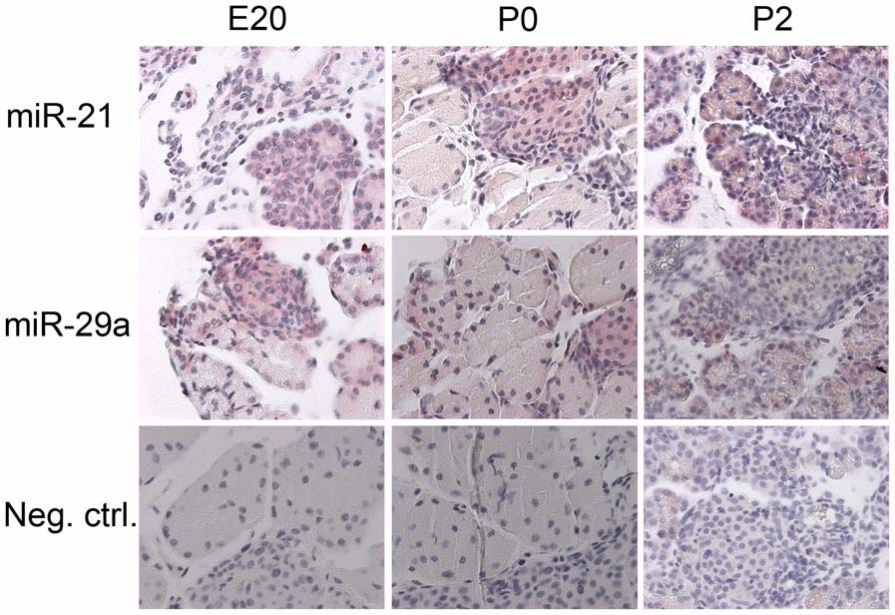
Localization of miR-21 and miR-29a in perintal rat pancreas at E20, P0 and P2 by in situ hybridization. Negative control: scrambled probe. Magnification: 400×.

ISH was used in order to identify the localization of the miRNA in pancreas according to exocrine or endocrine tissue (Table 1). Northern blots (Fig. 2) showed that miR-21, -29a, -23a, -141 and -125b-5p all were expressed in INS-1E cells and therefore expected to be localized to endocrine tissue. ISH confirmed the localization to endocrine and exocrine tissue (Fig. 3 and Fig.S3); however, miR-141 was only weakly expressed in endocrine cells and predominantly found at P0 and P2 in exocrine cells. Interestingly, at E20 most of the miRNAs (except miR-141) were expressed in both exocrine and endocrine tissue. MiR-29a was expressed in acinar and islet cells at E20 and changed to being mostly endocrine at P0 and P2. Localization of miR-21 changed from equally exocrine and endocrine at E20 to mostly exocrine expression at P2. MiR-23a and miR-125b-5p were mostly acinar, and expressed at a lower level in islets. MiR-376a, -376b-3p and -451 were localized to exocrine cells (miR-451 only at E20). Even though miR-376a, -376b-3p and -451 were not detected in INS-1E cells, they were detected in some islet cells at E20 and P0. Generally, the expression patterns identified in the array and northern hybridizations were also detected using ISH.

**Table 1:** Exocrine and endocrine localization and approximate levels of differentially regulated miRNAs in perinatal rat pancreas at E20, P0 and P2 determined using ISH.

MiRNA	E20		P0		P2	
	Exocrine	Endocrine	Exocrine	Endocrine	Exocrine	Endocrine
miR-21	+	+	(+)	+	++	(+)
miR-29a	+	+	+	++	+	++
miR-125b-5p	++	+	++	+	+	+
miR-23a	++	++	++	+	++	+
miR-376a	+	(+)	++	(+)	+	−
miR-141	−	−	+	−	+	(+)
miR-376b-3p	++	(+)	+	(+)	−	−
miR-451	++	+	−	−	−	−

‘+’ denotes miRNA present, ‘++’ denotes higher amount of miRNA present, (+) denotes presence of miRNA in some cells, ‘−’ denotes no miRNA present. MiR-21 showed heterogeneous staining between pancreata.

### Predicted target mRNAs of differentially regulated miRNAs

Prediction of miRNA target mRNAs was carried out using the algorithm miRanda [28], which predicted hundreds of potential targets for each miRNA. We limited the search to predicted mRNA targets, which were expressed and regulated in perinatal pancreas. 1057 genes were found to be differentially regulated in rat pancreas between E20 and P2, and 778 were fully annotated (L.W. Gaarn and J.H. Nielsen, unpublished data) of which 126 genes were potential miRNA targets in perinatal pancreas (Table S4). There were no mRNAs, which were targets of all differentially expressed miRNAs (Table S4). Since the list only contained miRNA targets regulated at the mRNA level it favored miRNAs altering mRNA transcript levels.

### Predicted biological functions of target mRNAs of regulated miRNAs

Pathway analysis using the 126 perinatally regulated genes that were also potential miRNA targets showed that the most significant Biological Processes (GO∶BP) category (using GeneCodis ver.2 [22], [29]) was ‘metabolic processes’ containing 16 genes. More specific categories were ‘response to hypoxia’, ‘lipid metabolic process’, ‘cholesterol metabolic process’, ‘cholesterol biosynthetic process’, ‘steroid metabolic process’, ‘isoprenoid or terpenoid biosynthetic process’ and ‘fatty acid oxidation’ each containing from 3–5 genes (Table 2 and Table S2). The identified KEGG pathways were ‘Valine, leucine and isoleucine degradation and synthesis’, ‘short chain fatty acid metabolism’, ‘pyruvate metabolism’ and ‘biosynthesis of steroids’. These pathways contain to a large degree the same mRNAs as the significant GO categories (Table S2).

**Table 2:** Selected biological processes in which predicted miRNA target genes are over-represented in perinatal rat pancreas according to Gene Ontology categories.

Annotations	Number of genes	Corrected p-value	Genes	MiRNAs
Lipid metabolic process	5	0.005	Soat1, Hdlbp, Acaca, Crot, Srebf1	miR-141/125b-5p,miR-451/125b-5p,miR-376a/376b-3p,miR-376b-3p,miR-21
Cholesterol metabolic process	5	1.7E-04	Soat1, Hdlbp, Sqle, Srebf1, Nsdhl	miR-141/125b-5p,miR-451/125b-5p,miR-21,miR-21,miR-376a/376b-3p
Cholesterol biosynthetic process	4	2.1E-04	Hmgcs1, Hmgcs2, Idi1, Nsdhl	miR-451,miR-21/376a/451,miR-376a/451,miR-376a/376b-3p
Isoprenoid biosynthetic process	4	0.002	Hmgcs1, Hmgcs2, Hmgcr, Idi1	miR-451,miR-21/376a/451,miR-29a,miR-376a/451
Steroid metabolic process	3	0.02	Soat1, Hdlbp, Srebf1	miR-141/125b-5p,miR-451/125b-5p,miR-21

**Figure 4:**
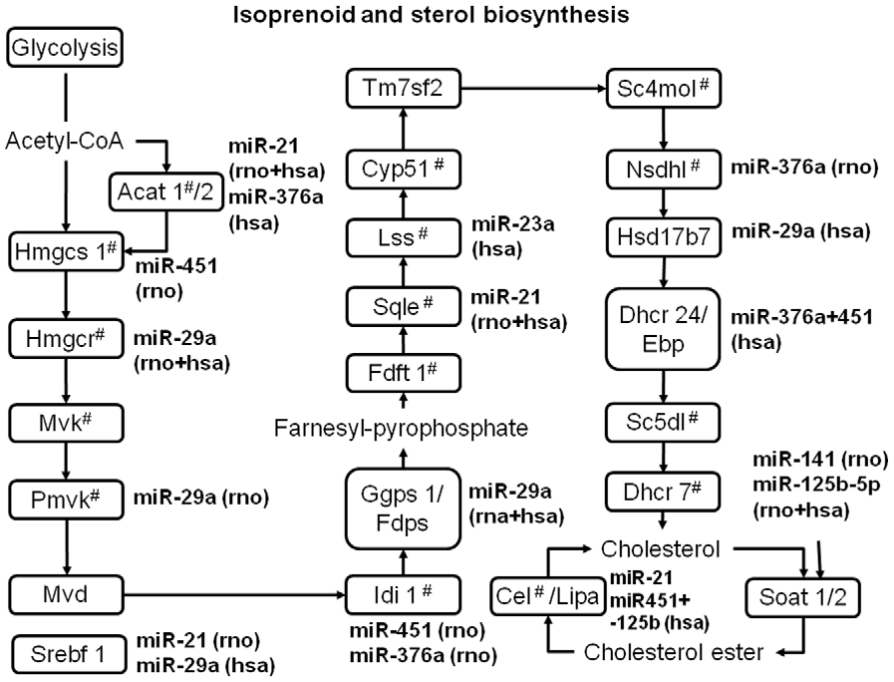
Enzyme pathway of isoprenoid/terpenoid and sterol biosynthesis adapted from KEGG [**21**] with indication of predicted target genes of regulated miRNAs in perinatal rat pancreas. SREBF1 targets are indicated with ‘#’ next to the name and are identified through Bennett *et al.* (2008) and Seo *et al.* (2009) [48], [49]. Abbreviations: *Hmgcs1* (3-hydroxy-3-methylglutaryl-Coenzyme A synthase 1 (soluble), *Hmgcr* (3-hydroxy-3-methylglutaryl-coA reductase), *Mvk* (mevalonate kinase), *Pmvk* (phosphomevalonate kinase), *Mvd* (mevalonate (diphospho) decarboxylase), *Idi1* (isopentenyl-diphosphate delta isomerase 1), *Fdps* (farnesyl diphosphate synthase), *Ggps1* (geranylgeranyl pyrophosphate synthetase), *Fdft1* (farnesyl diphosphate farnesyl transferase 1), *Lss* (lanosterol synthase), *Cyp51* (cytochrome P450, subfamily 51), *Tm7sf2* (transmembrane 7 superfamily member 2), *Sc4mol* (sterol-C4-methyl oxidase-like), *Nsdhl1* (NAD(P) dependent steroid dehydrogenase-like), *Hsd17b7*(hydroxysteroid (17-beta) dehydrogenase 7), *Hsdl2* (hydroxysteroid dehydrogenase like 2), *Dhcr24* (24-dehydrocholesterol reductase), *Ebp* (emopamil binding protein (sterol isomerase)), *Sc5dl* (sterol-C5-desaturase), *Dhcr7* (7-dehydrocholesterol reductase), *Soat1* (sterol O-acyltransferase 1), *Cel* (cholesterol ester lipase), rno (*rattus norvegicus*), hsa (*homo sapiens*).

**Figure 5:**
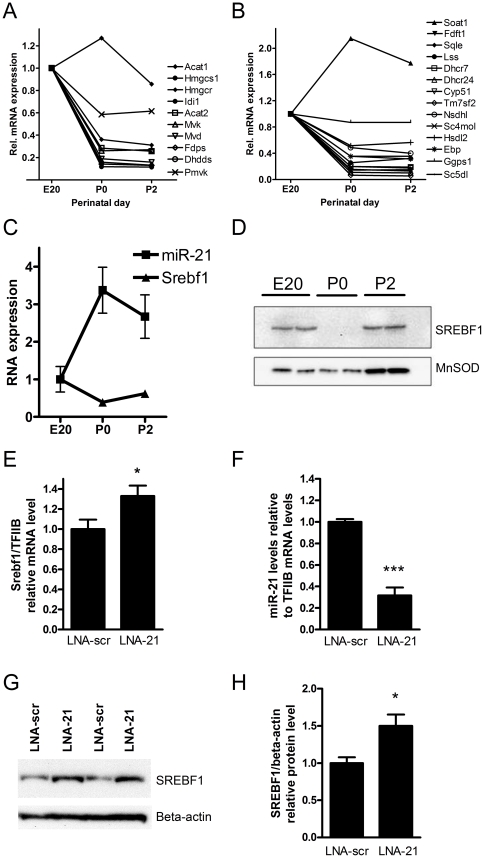
Correlations between mRNAs and miRNA-21 expression. Expression of mRNAs involved in isoprenoid (**A**) and cholesterol (**B**) biosynthesis in perinatal rat pancreas based on data from micro-array hybridizations. Data are presented as relative to the hybridization signal at E20. **C**. miR-21 and *Srebf1* mRNA levels in perinatal rat pancreas (n = 3). Endogenous control: *Rpl13alpha*. **D**. Western blot of SREBF1 in perinatal rat pancreas at E20, P0 and P2. Loading control: MnSOD. **E**. mRNA levels for *Srebf1* following knock-down of miR-21 in INS-1E cells (LNA-21: Knock-down of miR-21, LNA-scr: Scrambled control LNA, endogenous control: *TfIIB*). Data from 4 individual experiments each performed in duplicate nucleofections, *: p<0.05. **F**. miR-21 levels in INS-1E cells nucleofected with either knock-down LNA-oligonucleotide directed against miR-21 (LNA-21) or with a scrambled negative control LNA-oligonucleotide (LNA-scr). MiR-21 levels were determined using Q-PCR and are presented relative to the endogenous control *TfIIB*. Data from 3 individual experiments each performed in duplicate nucleofections, ***: p<0.001. **G** and **H**. Protein levels of SREBF1 following knock-down of miR-21 in INS-1E cells. Representative western blot of SREBF1 in INS-1E is shown in **G** and in panel **H** is shown the quantification of 5 blots containing samples from 9 individual nucleofections. (LNA-21: Knock-down of miR-21, LNA-scr: Scrambled control LNA, Loading control: beta-actin).*: p<0.05. Abbreviations: *Hmgcs1* (3-hydroxy-3-methylglutaryl-Coenzyme A synthase 1 (soluble)), *Hmgcr* (3-hydroxy-3-methylglutaryl-coA reductase), *Idi1* (isopentenyl-diphosphate delta isomerase 1), *Mvk* (mevalonate kinase), *Mvd* (mevalonate (diphospho) decarboxylase), *Fdps* (farnesyl diphosphate synthase), *Pmvk* (phosphomevalonate kinase), *Soat1* (sterol O-acyltransferase 1), *Fdft1* (farnesyl diphosphate farnesyl transferase 1), *Lss* (lanosterol synthase), *Dhcr7* (7-dehydrocholesterol reductase), *Dhcr24* (24-dehydrocholesterol reductase), *Cyp51* (cytochrome P450, subfamily 51), *Tm7sf2* (transmembrane 7 superfamily member 2), *Nsdhl1* (NAD(P) dependent steroid dehydrogenase-like), *Sc4mol* (sterol-C4-methyl oxidase-like), *Hsdl2* (hydroxysteroid dehydrogenase like 2), *Ebp* (emopamil binding protein (sterol isomerase)), *Ggps1* (geranylgeranyl pyrophosphate synthetase).

The order of the gene in the ‘Genes’ column corresponds to the order of the listed miRNA binding it in the ‘MiRNAs’ column. Where multiple miRNAs bind the same mRNA these are separated by slashes. For a full list of significant biological processes containing predicted miRNA target genes please consult with Table S2. Full names of genes may be found in Table S4 or in the legend to Figure 4 or Figure 5.

Thus, pathways related to lipid or specifically sterol synthesis, metabolism or degradation resulted from the pathway analysis. SREBF1, which is a key transcriptional regulator for genes involved in the early (isoprenoid biosynthesis) and late steps of cholesterol synthesis (Fig. 4) is a predicted target of miR-21. In addition, SQLE (squalene epoxidase), which catalyzes a rate-limiting step in sterol synthesis and mitochondrial ACAT1 (acetyl-coenzyme A acetyltransferase 1), which catalyzes the first step of isoprenoid biosynthesis, are also predicted targets of miR-21. Many genes involved in both early and late steps of cholesterol synthesis decrease their mRNA expression dramatically after birth (Fig. 5A, B). Among these are a number of predicted target mRNAs, such as *Acat1*, *Hmgcs1*, *Hmgcr*, *Pmvk*, *Idi1*, *Ggps1*, *Sqle*, *Nsdhl*, *Soat2*, *Cel*, *Hdlbp*, *Srebf1* (Fig. 5A, B, Fig. 4, Table S4).

### SREBF1 mRNA and protein levels in response to miR-21 levels

Expression of most of the mRNAs in the isoprenoid and sterol synthesis pathway decreased between E20 and P0 (Fig. 5A, B). SREBP1 mRNA and protein levels in the perinatal pancreas also showed a decrease between E20 and P0 and an increase in miR-21 of which *Srebf1* mRNA is a predicted target (Fig. 5C, D). Although both mRNA and protein levels of SREBP1 decreased sharply following miR-21 up-regulation at P0 (Fig. 5C, D), protein levels rose again at P2, where miR-21 continues to be expressed. Clearly, miR-21 cannot be the only factor controlling SREBP1 protein levels; also dietary lipids, which are known to affect SREBP1 levels and activity, change after birth.

To identify whether *Srebf1* could be a true target mRNA of miR-21 we introduced miR-21inhibitor (LNA-21) and control oligonucleotides (LNA-scr) into INS-1E cells, and extracted RNA and protein from these. MessengerRNA levels of *Srebf1* increased by about 30% after miR-21 knock-down (Fig. 5E). Also SREBP1 protein levels increased approximately 50% after miR-21 knock-down compared with a LNA-scr (Fig. 5G, H). These data were consistent with *Srebf1* being a target gene of miR-21. We also investigated whether mRNA and protein levels of SQLE changed when miR-21 levels were modified; however, mRNA levels were unaltered (data not shown) and we were unable to produce specific western blots using the available commercial SQLE antibody.

### Reporter-gene analysis of predicted miR-21 target genes

**Figure 6:**
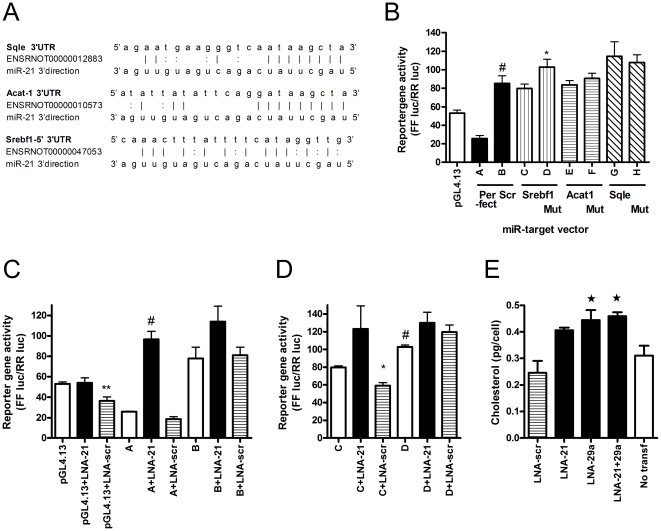
Reporter-gene analysis of predicted target genes of miR-21 and their binding sites. **A**. Predicted binding sites in the *Srebf1*, *Acat1* and *Sqle* 3′UTRs with suggested miR-21 binding. The p-values of the miRanda prediction algorithm for these three targets were: *Srebf1*: p = 0.0006, *Acat1*: p = 0.0004 and S*qle*: p = 0.001. **B–E**. Luciferase activities of reporter vectors normalized to pRL activity. **B**. Activities of target site and mutant site reporter vectors together with activity of pGL4.13. (The key for clone names and their target site is: A: Perfect match for miR-21, B: Scrambled miR-21 perfect match site, C: miR-21 *Srebf1* site, D: miR-21 *Srebf1* site with 2 bases mutated in seed sequence, E: miR-21 *Acat1* site, F: miR-21 *Acat1* site with 2 bases mutated in seed sequence, G: miR-21 *Sqle* site with 2 bases mutated in seed sequence.) #: p<0.0001 vs. ‘A’ (‘Perfect’), *: p<0.05 vs. ‘C’ (*Srebf1*). **C**. Luciferase activities after co-transfecting LNA-21 and LNA-scr with Perfect (‘A’) and Scrambled (‘B’) vectors, **: p<0.01 vs. ‘pGL4.13+LNA-21’, #: p<0.0001 vs. ‘A+LNA-scr’. **D**. Luciferase activities of the *Srebf1* target site (‘C’) and the 2 base mutated vector (‘D’) with co-transfection of LNA-21 and LNA-scr. #: p<0.0001 vs. ‘C’, *: p<0.05 vs. ‘C+LNA-21’. **E**. Total cholesterol levels of INS-1E cells following knock-down of miR-21 and/or miR-29a in INS-1E cells (LNA-21: Knock-down of miR-21, LNA-29a: Knock-down of miR-29a, LNA-scr: Scrambled control LNA, Untransf: Untransfected). *: p<0.05 vs. LNA-scr. The experiment was repeated 3 times.

To validate the predicted miR-21 target sites in the 3′UTR of *Srebf1*, *Acat1* and *Sqle* luciferase reporter vectors were constructed (Fig. 6A). Wild type target reporter constructs were pair-wise compared with mutant constructs in which 2 bases of the seed sequence in the miRNA binding site were changed. In order to validate the reporter assay a perfect miR-21 complementary target site sequence clone (A-construct) and a scrambled miR-21 target site sequence clone (B-construct) were transfected into INS-1E cells, which express mature miR-21 (Fig. 2). The luciferase activity of the ‘Perfect’ A-clone was lower than the ‘Scr’ B-clone (p<0.0001)(Fig. 6B). The B-construct had increased activity compared with pGL4.13, which could be due to destruction of a negative regulatory signal by the cloned insertion. Mutation of the miR-21 target site of *Srebf1* led to an increased luciferase activity (Fig. 6B, ‘C’ vs. ‘D’, p<0.0001). However, there were equal activities of the *Acat1* and S*qle* target clones and their corresponding mutant vectors (Fig. 6B, ‘E’ vs. ‘F’ and ‘G’ vs. ‘H’).

To test the direct responsiveness toward miR-21, miR-21 inhibitor (LNA-21) and negative control (LNA-scr) were co-transfected with the reporter vectors (Fig. 6D, Fig. S4A, B). As control pGL4.13, ‘Perfect’ and ‘Scr’ vectors were also compared (Fig. 6C). Removal of miR-21 from pGL4.13 did not have any effect indicating that this contains no endogenous miR-21 site. There was a slight inhibitory action of LNA-scr when co-transfected with pGL4.13 (Fig. 6C, ‘pGL4.13’ vs. ‘pGL4.13+LNA-scr’). Co-transfection of miR-21 inhibitor caused a 3-fold increase in the luciferase activity of the ‘Perfect’ binding site vector (‘A+LNA-scr’ vs ‘A+LNA-21’, p<0.0001), demonstrating effective inhibition of miR-21 by the LNA-oligonucleotide and confirming the Q-PCR assay of miR-21 shown in Fig. 5F.

Inhibiting miR-21 increased luciferase activity of the *Srebf1* target vector (Fig. 6D ‘C+LNA-21’ vs. ‘C+LNA-scr’, p<0.05), demonstrating that the target site is functional, but the quantitative effect of removing cellular miR-21 is small. Removing miR-21 had no effect on the mutant *Srebf1* target site construct.

Reporter-gene analysis of the predicted target sites of *Acat1* and *Sqle* were unable to confirm that these two predicted targets of miR-21 were functional (Fig. 6 and Fig. 4A and 4B), which is consistent with measurements of mRNA levels. The transfection efficiency of the LNA-spiked oligonucleotides is about 50% in INS-1E cells, whereas the plasmid transfections efficiency is 25% (data not shown). Most cells transfected with reporter plasmids were therefore also transfected with LNA-spiked oligonucleotides. In summary, out of the three tested targets, *Srebf1* was conclusively found to be a functional target of miR-21.

### Analysis of cholesterol levels

To determine whether the presence of multiple targets of miR-21 and miR-29a (Fig. 4) along the cholesterol synthesis pathway resulted in a cumulative effect on cholesterol levels, we extracted and measured cholesterol in INS-1E cells nucleofected with oligonucleotide inhibitors of miR-21 and miR-29a (LNA-21 and LNA-29a) and negative control oligonucleotide (LNA-scr). Cells with inhibited miR-29a alone or with miR-21 had increased total cholesterol levels compared with LNA-scr (Fig. 6E, ‘LNA-scr’ vs. ‘LNA-29a’ or vs. ‘LNA-21+29a’, p<0.05). There was an insignificant increase in cholesterol levels of cells treated with miR-21 inhibitor. Thus, inhibition of miR-21 and miR-29a simultaneously and miR-29a by itself increased total cholesterol levels, showing that the increase of miR-21 and miR-29a following birth is likely to functionally participate in the decrease of the cholesterol synthesis pathway.

## Discussion

This study represents the first characterization of miRNA expression in perinatal rat pancreas. We have 1) Determined the miRNA profile of perinatal pancreas, which is undergoing large structural and metabolic changes in the period around birth and identified and validated 7 miRNAs regulated more than 1.5-fold from E20 to P2; 2) Localized regulated miRNAs by ISH; 3) Performed focused pathway analysis of pathways affected by regulated miRNAs using predicted mRNA target genes also regulated in perinatal pancreas; 4) Shown that biochemical pathways relating to sterol and lipid biosynthesis and metabolism are affected by the perinatally regulated miRNAs; 5) Validated *Srebf1* as target gene of miR-21 and shown an effect of miR-21 and miR-29a on INS-1E beta-cell cholesterol levels.

These observations contribute to the understanding of the structural and functional changes occurring in perinatal pancreas and further our understanding of how beta-cells proliferate, differentiate and functionally mature, as these are major events taking place in pancreas around time of birth. The rate of beta-cell proliferation in neonatal pancreas is approximately 10–30 fold greater than in adult pancreas [15], [30]. Furthermore, during the perinatal period the structure of islets changes from small islets and scattered beta-cells to more organized, mature islets. In rat pancreas, there is a marked increase in amount of beta-cells between E20 to P0 and further to P2 [15], [30]. The function of the pancreas also changes during this period, where there is a transition from receiving nutrients via the placenta to ingestion of milk.

Seven miRNAs were differentially regulated more than 1.5-fold in the perinatal pancreas. None of these were highly expressed and the general fold regulation is quite low. However, miR-21 and miR-29a were 2.5- and 4-fold increased. MiR-21 has been found to be up-regulated in several studies of pancreas cancer, is associated with a poor prognosis and generally increases cellular proliferation [31]–[33]. MiR-21 up-regulation coincides with the rise in beta-cell proliferation and could be a contributing factor to this. ISH was used to assess miR-21 expression in human pancreatic adenocarcinoma, where miR-21 expression was found in malignant tissue but not in normal acinar tissue. In our ISH study we localized miR-21 to be expressed in perinatal acinar cells and in pancreatic islets, correlating well with the rapid proliferation in exocrine and endocrine cells of postnatal pancreas.

MiR-29a has previously been recognized as a miRNA increased in adipose tissue and skeletal muscle in diabetes [34], [35]. Here, miR-29a was expressed in exocrine and endocrine pancreas with highest expression in islets, in line with findings from adult pancreas [12]. MiR-29 family members are often down-regulated in cancer and forced expression of miR-29a is reported to reduce proliferation and invasiveness presumably via CDC42 and p85alpha dependent up-regulation of p53 [36], [37]. MiR-125b-5p [38] as well as miR-23a and miR-376a are up-regulated in pancreatic cancer [32], [39]. MiR-23a has been reported to be expressed in endothelial cells [40], but our ISH data do not reveal miR-23a expression in neither small nor large vessels in perinatal pancreas. It should be noted that although our results show the distribution of miRNAs between the exocrine and the endocrine compartment of perinatal pancreas, they do not reveal the specific localization of miRNAs within islets to i.e. alpha or beta-cells.

In general, we observe a similar expression profile by northern blotting as initially observed by array hybridizations. There are three exceptions to this: First, array hybridization show that miR-376a is increased at P0 compared with E20, whereas northern blots showed a decrease in mature miR-376a at P0. Although ISH is not quantitative this method confirmed the northern blots, since we generally detected a lower signal of miR-376a at P0 and P2 than at E20. Secondly, array data showed differential regulation of miR-341; however, the mature miR-341 species was not detected on multiple northern blots, whereas high signal intensities were observed for pri-miR-341 or pre-miR-341 (Fig.S2). Thus, the LNA-modified oligo-arrays were accurate, but verification by another method is necessary. We favored northern blotting over Q-PCR, because the biologically inactive precursor species may be co-amplified with the mature species when performing stem-loop RT-PCR [41]. Third, the regulation of miR-23a measured using northern blotting was below 1.5-fold change, but as it was slightly increased also by northern blotting, we included it in the further studies.

The mRNA expression profile of perinatal rat pancreas was used in order to enrich the pathway analysis for genuine targets of regulated miRNAs. This procedure focuses on miRNA targets regulated at the mRNA level and disfavors miRNA-mRNA interactions that primarily result in translational inhibition.

The pathway analysis identified cholesterol or lipid metabolism as pathways selectively targeted by the perinatally regulated miRNAs (Fig. 4). One of these is *Srebf1* (or *Srebp1*), which together with *Srebf2* is responsible for activation of genes involved in cholesterol and lipid metabolism, when exogenous cholesterol levels are low. The miR-21 binding site of *Srebf1* is rat specific; however the human *SREBF1* gene contains a binding site for miR-29a, and this miRNA is also increased in islets at P0 and P2 and has an effect on cholesterol levels. Thus, also human sterol synthesis is predicted to be selectively targeted by the miRNAs identified as being regulated from E20 to P2 in rat pancreas (Fig. 4). Whether this miRNA expression pattern is conserved in human fetal and newborn pancreas needs to be determined.

*SREBP1* is important for regulation of glucose-stimulated insulin secretion of adult beta-cells. Over-expression of *SREBP1* decreases glucose-stimulated insulin secretion, and SREBP1 is activated in beta-cell gluco-lipotoxicity [42]–[44]. In the clonal INS-1E beta-cells SREBP1 and miR-21 are co-expressed, however, co-localization was not determined in perinatal beta-cells. Cholesterol levels in pancreatic beta-cells are tightly regulated as too much cholesterol will inhibit glucose-stimulated insulin secretion and induce lipotoxicity, whereas too little cholesterol impairs membrane fluidity and by this mechanism decreases insulin exocytosis [45], [46]. It is possible that up-regulation of miR-21 and/or miR-29a in perinatal rat pancreas act through down-regulation of *Srebf1* and other genes in the cholesterol synthesis pathway to fine-tune cholesterol levels and promote functional maturation of beta-cells following birth.

Whether the perinatal regulation of miR-21 and miR-29a is present in other tissues remain to be investigated; however, cholesterol synthesis of rat liver, intestines and brain decrease sharply at the day of birth and synthesis rates then increase again on postnatal day 2 [47], which is consistent with the mRNA and miRNA expression pattern observed in pancreas.

The quantitative effect of miR-21 on *Srebf1* was small as determined from western blots and luciferase reporter assays. However, we believe that our assays genuinely capture the effects of these miRNAs, because we have shown effective inhibition of miR-21 using LNA-modified oligonucleotides (Fig. 5F) and the effects of the functional reporter assays on the *Srebf1* target site (Fig. 6B and D) mirror the effects of miR-21 on *Srebf1* mRNA and protein levels (Fig. 5E and H). It seems likely that these small effects are functional given that we can increase cholesterol levels by inhibiting miR-21 and miR-29a.

In conclusion, a comparison between the mRNA and the miRNA expression pattern of perinatal pancreas showed that regulated miRNAs selectively target cholesterol synthetic genes. Specifically the up-regulated miR-21 mediated down-regulation of *Srebf1*.

## Supporting Information

Figure S1 **Microarray analysis of miRNAs expressed in the perinatal rat pancreas.** Heat-map showing a hierarchical gene-tree cluster of the 108 miRNAs that are expressed at E20, P0 and P2 with signal intensities >25.
(TIF)

Figure S2 **Validation of miRNA expression using northern blot.** Whole images of northern blot membranes. The mature miRNAs are marked with an arrow. E20 and P2 were loaded in triplicates and P0 in duplicates. Total RNA from INS-1E cells were used as a surrogate control for miRNA expression in beta-cells.
(TIF)

Figure S3 **Images from ISH sections stained for miR-21, -29a, -451, -141, -376a, -376b-3p, -23a, -125b-5p and corresponding scrambled control.** Magnification: 400×.
(TIF)

Figure S4 **Reporter-gene analysis of predicted target sites in** ***Acat1*** **and** ***Sqle*** **in response to exogenous miR-21.** **A.** Luciferase activities of the *Acat1* target site (‘E’) and the 2 base mutated vector (‘F’) with co-transfection of LNA-21 and LNA-scr. *: p<0.05 vs. ‘E’, #: p<0.005 vs. ‘F+LNA-21’. **B.** Luciferase activities of the *Sqle* target site (‘G’) and the 2 base mutated vector (‘H’) with co-transfection of LNA-21 and LNA-scr. Data are from 4 individual experiments each performed in duplicate transfections.
(TIF)

Table S1 **Quantification of northern blots and comparison with array hybridizations.**
(DOC)

Table S2 **Complete list of significant biological processes containing predicted miRNA target genes.** **A**. GO categories **B**. KEGG categories.
(DOC)

Table S3 **List of oligonucleotides used for Q-PCR and for cloning miR-21 target reporter vectors.**
(DOC)

Table S4 **Predicted target mRNAs of differentially expressed miRNAs.** List of the common genes predicted by Microcosm and regulated in the perinatal pancreas divided into clusters according to shared miRNAs.
(DOC)
