# Supplementary figures and images for: Expression and Localization of microRNAs in Perinatal Rat Pancreas: Role of miR-21 in Regulation of Cholesterol Metabolism
Source: PLoS One. 2011 Oct 11;6(10):e25997. doi: 10.1371/journal.pone.0025997 (PMC3191174; doi:10.1371/journal.pone.0025997)

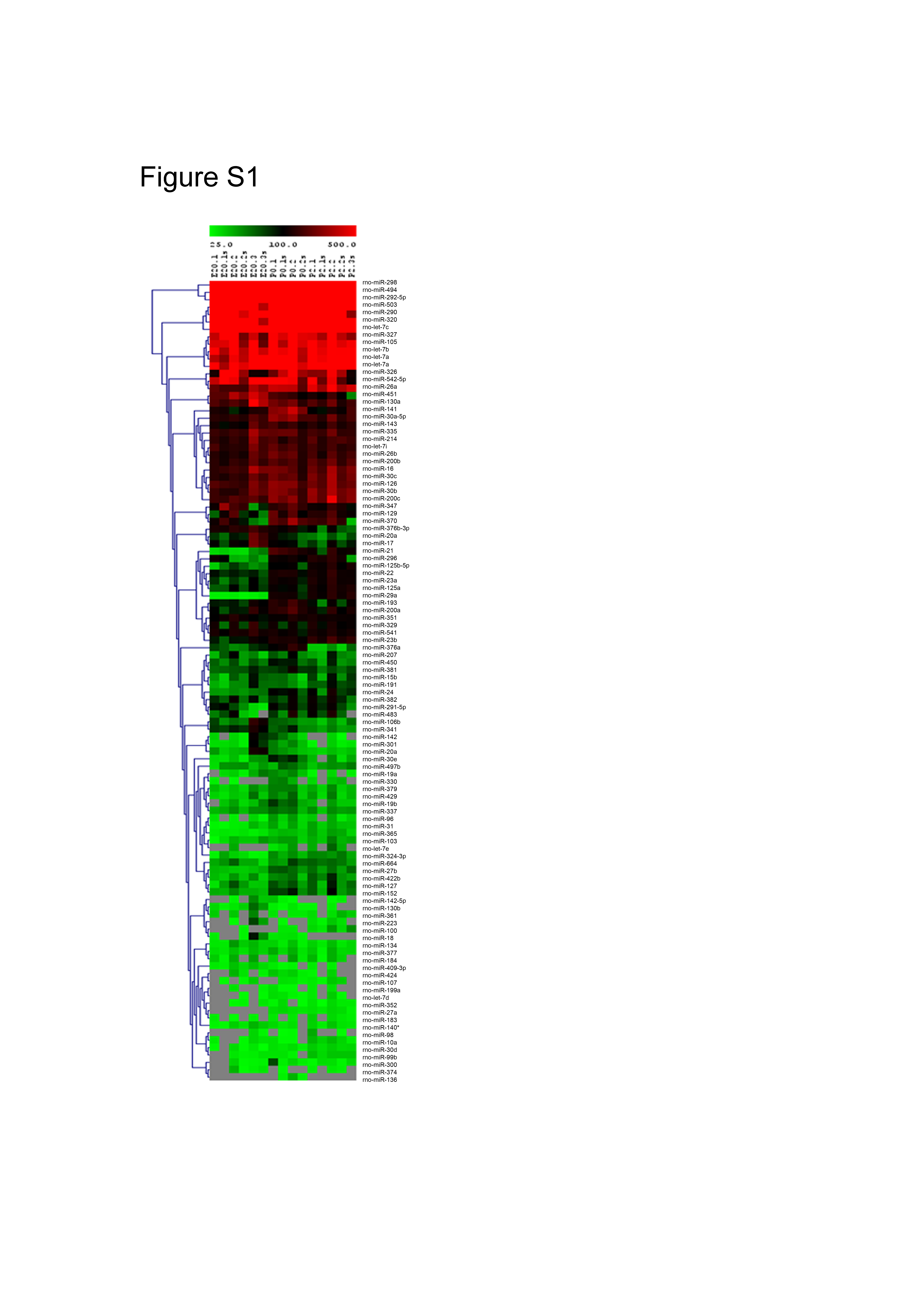

Supplement: Figure S1 — Microarray analysis of miRNAs expressed in the perinatal rat pancreas. Heat-map showing a hierarchical gene-tree cluster of the 108 miRNAs that are expressed at E20, P0 and P2 with signal intensities >25. (TIF) [file pone.0025997.s001.tif]

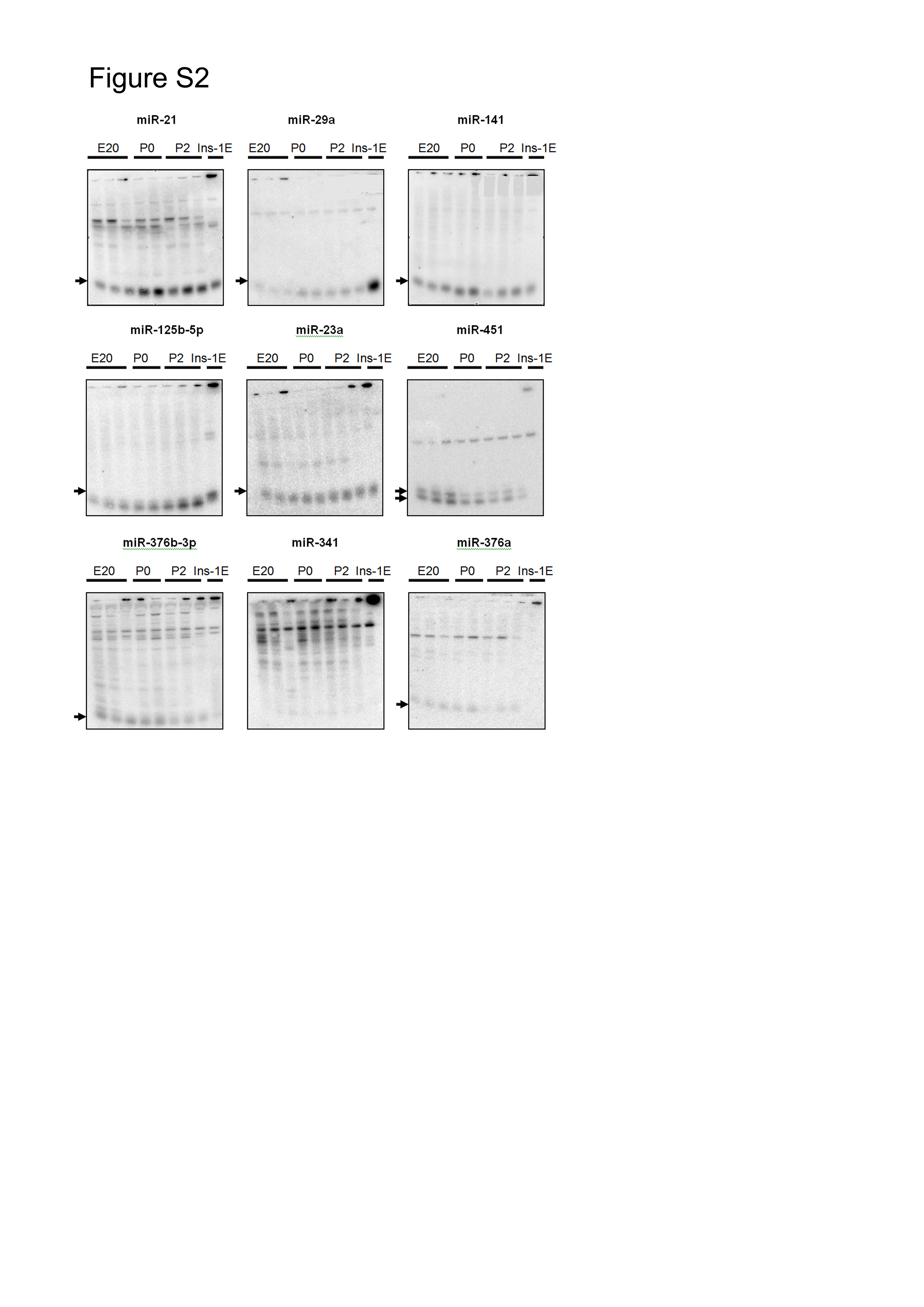

Supplement: Figure S2 — Validation of miRNA expression using northern blot. Whole images of northern blot membranes. The mature miRNAs are marked with an arrow. E20 and P2 were loaded in triplicates and P0 in duplicates. Total RNA from INS-1E cells were used as a surrogate control for miRNA expression in beta-cells. (TIF) [file pone.0025997.s002.tif]

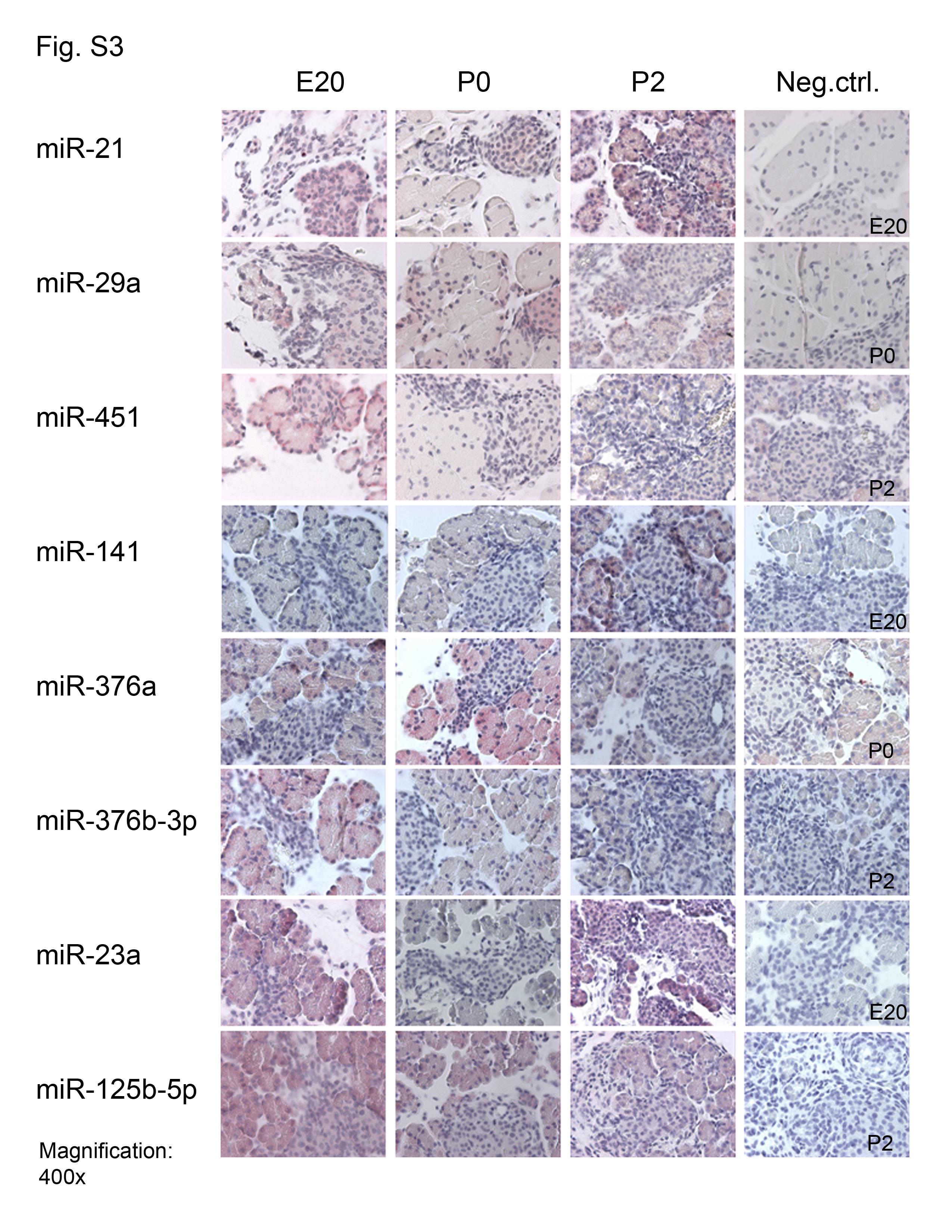

Supplement: Figure S3 — Images from ISH sections stained for miR-21, -29a, -451, -141, -376a, -376b-3p, -23a, -125b-5p and corresponding scrambled control. Magnification: 400×. (TIF) [file pone.0025997.s003.tif]
