# Supplementary material for: Expression and Localization of microRNAs in Perinatal Rat Pancreas: Role of miR-21 in Regulation of Cholesterol Metabolism
Source: PLoS One. 2011 Oct 11;6(10):e25997. doi: 10.1371/journal.pone.0025997 (PMC3191174; doi:10.1371/journal.pone.0025997)
Supplement: Table S1 — Quantification of northern blots and comparison with array hybridizations. (DOC) [file pone.0025997.s005.doc]

**Supplemental Table S1: Normalized miRNA hybridization signals from arrays and northern blots.**

|  |  | **Array** | | | **Northern blot** | | |
| --- | --- | --- | --- | --- | --- | --- | --- |
| **Cluster** | **miRNA** | **E20** | **P0** | **P2** | **E20** | **P0** | **P2** |
| I | miR-21 | 1.0±0.3 | 3.1±0.8 | 2.4±0.5 | 1.0±0.3 | 3.4±0.6 | 2.7±0.6 |
|  | miR-29a | 1.0±0.1 | 3.7±1.0 | 4.0±0.9 | 1.0±0.1 | 2.0±0.2 | 1.9±0.2 |
| II | miR-23a | 1.0±0.2 | 1.3±0.3 | 1.6±0.3 | 1.0±0.03 | 1.1±0.1 | 1.1±0.1 |
|  | miR-125b-5p | 1.0±0.2 | 1.1±0.1 | 1.6±0.2 | 1.0±0.5 | 2.1±0.5 | 4.8±1.4 |
| III | miR-141 | 1.0±0.2 | 2.1±0.4 | 1.0±0.3 | 1.0±0.2 | 1.9±0.3 | 1.0±0.4 |
|  | miR-376a | 1.0±0.2 | 1.5±0.3 | 0.6±0.3 | 1.0±0.1 | 0.8±0.1 | 0.5±0.1 |
| IV | miR-376b-3p | 1.0±0.2 | 0.7±0.1 | 0.5±0.2 | 1.0±0.3 | 1.0±0.1 | 0.3±0.1 |
|  | miR-451 | 1.0±0.3 | 0.5±0.2 | 0.4±0.2 | 1.0±0.2 | 0.5±0.02 | 0.3±0.1 |
|  | miR-341 | 1.0±0.2 | 0.8±0.3 | 0.6±0.1 | ND | ND | ND |

Data are mean±SD. All hybridization results are normalized to the expression at E20. ND: Not determined.
