# Supplementary material for: Expression and Localization of microRNAs in Perinatal Rat Pancreas: Role of miR-21 in Regulation of Cholesterol Metabolism
Source: PLoS One. 2011 Oct 11;6(10):e25997. doi: 10.1371/journal.pone.0025997 (PMC3191174; doi:10.1371/journal.pone.0025997)
Supplement: Table S2 — Complete list of significant biological processes containing predicted miRNA target genes. A. GO categories B. KEGG categories. (DOC) [file pone.0025997.s006.doc]

**Supplemental Table S2: Functional annotations of potential miRNA regulated genes in the perinatal pancreas according to GO and KEGG**.

| **GO: Biological Processes (BP)** |  |  |
| --- | --- | --- |
| **Annotations** | **Number of genes** | **Corrected p-value** |
| metabolic processes | 16 | 8.5E-10 |
| signal transduction | 7 | 0.04 |
| regulation of transcription, DNA-dependent | 7 | 0.04 |
| oxidation reduction | 7 | 0.02 |
| cholesterol metabolic process | 5 | 1.4E-04 |
| lipid metabolic process | 5 | 0.005 |
| response to hypoxia | 5 | 0.02 |
| response to drug | 5 | 0.04 |
| proteolysis | 5 | 0.04 |
| transcription | 5 | 0.04 |
| transport | 5 | 0.04 |
| cholesterol biosynthetic process | 4 | 2.1E-04 |
| angiogenesis | 4 | 0.02 |
| carbohydrate metabolic process | 4 | 0.03 |
| negative regulation of apoptosis | 4 | 0.04 |
| isoprenoid biosynthetic process | 3 | 0.002 |
| ATP biosynthetic process | 3 | 0.01 |
| steroid metabolic process | 3 | 0.02 |
| liver development | 3 | 0.02 |
| intracellular protein transport | 3 | 0.04 |
| in utero embryonic development | 3 | 0.04 |
| heart development | 3 | 0.04 |
| negative regulation of transcription from RNA polymerase II promoter | 3 | 0.04 |
| hypoxanthine biosynthetic process | 2 | 0.001 |
| positive regulation of exocytosis | 2 | 0.004 |
| protein export from nucleus | 2 | 0.02 |
| tissue remodeling | 2 | 0.02 |
| negative regulation of catalytic activity | 2 | 0.02 |
| regulation of calcium ion transport | 2 | 0.03 |
| glucose homeostasis | 2 | <0.05 |
| glycolysis | 2 | <0.05 |
| response to organic nitrogen | 2 | 0.04 |
| Wnt receptor signaling pathway | 2 | <0.05 |
| cation transport | 2 | <0.05 |
| cellular calcium ion homeostasis | 2 | <0.05 |
| lipid catabolic process | 2 | <0.05 |
| fatty acid metabolic process | 2 | <0.05 |
| male gonad development (BP) | 2 | 0.03 |
| positive regulation of specific transcription from RNA polymerase II promoter (BP) | 2 | 0.04 |

Total number of input genes = 126

| **KEGG pathways** |  |  |
| --- | --- | --- |
| **Annotations** | **Number of genes** | **Corrected p-value** |
| valine, leucine and isoleucine degradation | 8 | 7.7E-10 |
| butanoate metabolism | 5 | 9.3E-06 |
| biosynthesis of steroids | 4 | 4.2E-05 |
| pyruvate metabolism | 4 | 1.7E-04 |
| wnt signaling pathway | 4 | 0.02 |
| calcium signaling pathway | 4 | 0.03 |
| synthesis and degradation of ketone bodies | 3 | 5.5E-05 |
| valine, leucine and isoleucine biosynthesis | 3 | 1.1E-04 |
| propanoate metabolism | 3 | 0.003 |
| Melanogenesis | 3 | 0.03 |
| insulin signaling pathway | 3 | <0.05 |
| terpenoid biosynthesis | 2 | 0.001 |
| histidine metabolism | 2 | 0.03 |
| bile acid biosynthesis | 2 | 0.03 |
| starch and sucrose metabolism | 2 | 0.04 |
| n-glycan biosynthesis | 2 | <0.05 |
| lysine degradation | 2 | <0.05 |
| aminoacyl-trna biosynthesis | 2 | <0.05 |
| fatty acid metabolism | 2 | <0.05 |
| tryptophan metabolism | 2 | <0.05 |

Total number of input genes = 126
