# Supplementary material for: Expression and Localization of microRNAs in Perinatal Rat Pancreas: Role of miR-21 in Regulation of Cholesterol Metabolism
Source: PLoS One. 2011 Oct 11;6(10):e25997. doi: 10.1371/journal.pone.0025997 (PMC3191174; doi:10.1371/journal.pone.0025997)
Supplement: Table S3 — List of oligonucleotides used for Q-PCR and for cloning miR-21 target reporter vectors. (DOC) [file pone.0025997.s007.doc]

| **Gene/target** | **Oligo** | **Sequence** |
| --- | --- | --- |
| *Srebf1* mRNA (amplify 1a and 1c isoforms) | Sense | 5’cccctgggcctggaatcaaaga |
|  | Antisense | 5’gggtgttcccaggaagggtt |
| *Sqle* mRNA | Sense | 5’tgtctgcagcccggtggcta |
|  | Antisense | 5’gccatggtggaaagcaacccca |
| *Rpl13alpha* mRNA | Sense | 5’ccaccctatgacaagaaaaagc |
|  | Antisense | 5’acattcttttctgcctgtttcc |
| *TfIIB* mRNA | Sense | 5’gttctgctccaacctttgcct |
|  | Antisense | 5’tgtgtagctgccatctgcactt |
| Perfect | A sense | 5’ctagacagctgttacatcaacatcagtctgataagctaagaaaggccgg |
|  | A antisense | 5’cctttcttagcttatcagactgatgttgatgtaacagctgt |
| Scrambled | B sense | 5’ctagaactagtttacatcacataatctattcgaaatcgagaaaggccgg |
|  | B antisense | 5’cctttctcgatttcgaatagattatgtgatgtaaactagtt |
| *Srebf1* | C sense | 5’ctagagcatgcttacaaactttattttcataggttgagaaaggccgg |
|  | C antisense | 5’cctttctcaacctatgaaaataaagtttgtaagcatgct |
| *Srebf1* mut | D sense | 5’ctagaggtaccttacaaactttattttcatggattgagaaaggccgg |
|  | D antisense | 5’cctttctcaatccatgaaaataaagtttgtaaccatggt |
| *Acat1* | E sense | 5’ctagagagctcggccagattatattcaggataagctatttcaggccgg |
|  | E antisense | 5’cctgaaatagcttatcctgaatataatctggccgagctct |
| *Acat1* mut | F sense | 5’ctagagagctcggccagattatattcaggatcagatatttcaggccgg |
|  | F antisense | 5’cctgaaatatctgatcctgaatataatctggccgagctct |
| *Sqle* | G sense | 5’ctagactcgagttgagaatgaagggtcaataagctagatgtggccgg |
|  | G antisense | 5’ccacatctagcttattgacccttcattctcaactcgag t |
| *Sqle* mut | H sense | 5’ctagagctagcttgagaatgaagggtcaatcagatagatgtggccgg |
|  | H antisense | 5’ccacatctatctgattgacccttcattctcaagctagct |
| *miR-21* | Sense (fw) | 5’acactccagctgggtagcttatcagactga |
|  | Looped RT | 5’ctcaactggtgtcgtggagtcggcaattcagttgagtcaacatc |
|  | Universal antisense | 5’tggtgtcgtggagtcg |

**Table S3: List of oligonucleotides used for Q-PCR and for cloning of miRNA reporter vectors.**
