# Supplementary material for: Expression and Localization of microRNAs in Perinatal Rat Pancreas: Role of miR-21 in Regulation of Cholesterol Metabolism
Source: PLoS One. 2011 Oct 11;6(10):e25997. doi: 10.1371/journal.pone.0025997 (PMC3191174; doi:10.1371/journal.pone.0025997)
Supplement: Table S4 — Predicted target mRNAs of differentially expressed miRNAs. List of the common genes predicted by Microcosm and regulated in the perinatal pancreas divided into clusters according to shared miRNAs. (DOC) [file pone.0025997.s008.doc]

**Supplemental Table S4**

**MiRNA target genes predicted by MiRanda (MicroCosm) and regulated in the perinatal pancreas**

| **Cluster I** |  |  | |
| --- | --- | --- | --- |
| **MiRNA** | **Gene** | **Description** | |
| miR-29a | Ace2 | angiotensin I converting enzyme (peptidyl-dipeptidase A) 2 | |
|  | Angptl4 | angiopoietin-like 4 | |
|  | Cmas | cytidine monophosphate N-acetylneuraminic acid synthetase | |
|  | Csda | cold shock domain protein A | |
|  | Cspg4 | chondroitin sulfate proteoglycan 4 | |
|  | Cugbp1 | CUG triplet repeat, RNA binding protein 1 | |
|  | Herc4 | hect domain and RLD 4 | |
|  | Hmgcr | 3-hydroxy-3-methylglutaryl-Coenzyme A reductase | |
|  | Mad2l1bp | MAD2L1 binding protein | |
|  | Mgat4b | mannosyl (alpha-1,3-)-glycoprotein beta-1,4-N-acetylglucosaminyltransferase, isozyme B | |
|  | Narf | nuclear prelamin A recognition factor | |
|  | Plscr3 | phospholipid scramblase 3 | |
|  | Prps1 | phosphoribosyl pyrophosphate synthetase 1 | |
|  | Rmnd5a | required for meiotic nuclear division 5 homolog A (S. cerevisiae) | |
|  | Sdc2 | syndecan 2 | |
|  | Smpdl3a | sphingomyelin phosphodiesterase, acid-like 3A | |
|  | Stx1a | syntaxin 1A (brain) | |
|  | Tspan3 | tetraspanin 3 | |
|  | Yars | tyrosyl-tRNA synthetase | |
| miR-21 | Acat1 | acetyl-coenzyme A acetyltransferase 1 | |
|  | Atp1a1 | ATPase, Na+/K+ transporting, alpha 1 polypeptide | |
|  | Baiap2 | BAI1-associated protein 2 | |
|  | Bok | BCL2-related ovarian killer | |
|  | Cyhr1 | cysteine and histidine rich 1 | |
|  | Gbe1 | glucan (1,4-alpha-), branching enzyme 1 | |
|  | Gcs1 | glucosidase 1 | |
|  | Gtf2e2 | general transcription factor IIE, polypeptide 2, beta | |
|  | Hdc | histidine decarboxylase | |
|  | Hmgcs2 | 3-hydroxy-3-methylglutaryl-Coenzyme A synthase 2 (mitochondrial) | |
|  | Mccc2 | methylcrotonoyl-Coenzyme A carboxylase 2 (beta) | |
|  | Mrps28 | mitochondrial ribosomal protein S28 | |
|  | Plcb1 | phospholipase C, beta 1 (phosphoinositide-specific) | |
|  | Pxmp4 | peroxisomal membrane protein 4 | |
|  | Rexo2 | REX2, RNA exonuclease 2 homolog (S. cerevisiae) | |
|  | Rpp38 | ribonuclease P/MRP 38 subunit (human) | |
|  | Tmem77 | transmembrane protein 77 | |
|  | Sqle | squalene epoxidase | |
|  | Srebf1 | sterol regulatory element binding transcription factor 1 | |
|  | Ugp2 | UDP-glucose pyrophosphorylase 2 | |
| miR-29a + miR-21 | Xpo6 | exportin 6 | |
| **Cluster II** | **Gene** | **Description** | |
| miR-125b-5p | Abcf1 | ATP-binding cassette, sub-family F (GCN20), member 1 | |
|  | Acrbp | acrosin binding protein | |
|  | Aldh2 | aldehyde dehydrogenase 2 family (mitochondrial) | |
|  | Alkbh3 | alkB, alkylation repair homolog 3 (E. coli) | |
|  | Arsb | arylsulfatase B | |
|  | Bcat2 | branched chain aminotransferase 2, mitochondrial | |
|  | Cyhr1 | cysteine and histidine rich 1 | |
|  | Gcnt1 | glucosaminyl (N-acetyl) transferase 1, core 2 | |
|  | Hdc | histidine decarboxylase | |
|  | Hdlbp | high density lipoprotein binding protein (vigilin) | |
|  | Mamdc2 | MAM domain containing 2 | |
|  | Nudt22 | nudix (nucleoside diphosphate linked moiety X)-type motif 2 | |
|  | Pacsin3 | protein kinase C and casein kinase substrate in neurons 3 | |
|  | Plcb1 | phospholipase C, beta 1 (phosphoinositide-specific) | |
|  | Ppy | pancreatic polypeptide | |
|  | Rhov | ras homolog gene family, member V | |
|  | Slc35c2 | solute carrier family 35, member C2 | |
|  | Serpina6 | serine (or cysteine) peptidase inhibitor, clade A, member 6 | |
|  | Slit3 | slit homolog 3 (Drosophila) | |
|  | Soat1 | sterol O-acyltransferase 1 | |
|  | Sstr3 | somatostatin receptor 3 | |
|  | Tmem77 | transmembrane protein 77 | |
| miR-23a | Ada | adenosine deaminase | |
|  | Cand1 | cullin-associated and neddylation-dissociated 1 | |
|  | Cryl1 | crystallin, lambda 1 | |
|  | Ercc3 | excision repair cross-complementing rodent repair deficiency, complementation group 3 | |
|  | Hmgb2 | high mobility group box 2 | |
|  | Lgmn | Legumain | |
|  | Lmbrd1 | LMBR1 domain containing 1 | |
|  | Mccc1 | methylcrotonoyl-Coenzyme A carboxylase 1 (alpha) | |
|  | Pdhb | pyruvate dehydrogenase (lipoamide) beta | |
|  | Plcb4 | phospholipase C, beta 4 | |
|  | Pln | Phospholamban | |
|  | Rg9mtd2 | RNA (guanine-9-) methyltransferase domain containing 2 | |
|  | Rnf138 | ring finger protein 138 | |
|  | Tmem27 | transmembrane protein 27 | |
| **Cluster III** | **Gene** | | **Description** |
| miR-141 | Alg5 | | asparagine-linked glycosylation 5, dolichyl-phosphate beta-glucosyltransferase homolog (S. cerevisiae) |
|  | Ap1m1 | | adaptor-related protein complex 1, mu 1 subunit |
|  | Atp1b1 | | ATPase, Na+/K+ transporting, beta 1 polypeptide |
|  | Atp2c1 | | ATPase, Ca++ transporting, type 2C, member 1 |
|  | Cadps2 | | Ca++-dependent secretion activator 2 |
|  | Cmas | | cytidine monophosphate N-acetylneuraminic acid synthetase |
|  | Cryl1 | | crystallin, lambda 1 |
|  | Gbe1 | | glucan (1,4-alpha-), branching enzyme 1 |
|  | Gc | | group specific component |
|  | Gcs1 | | glucosidase 1 |
|  | Mrpl17 | | mitochondrial ribosomal protein L17 |
|  | Ptger3 | | prostaglandin E receptor 3 (subtype EP3) |
|  | Rg9mtd2 | | RNA (guanine-9-) methyltransferase domain containing 2 |
|  | Rnf138 | | ring finger protein 138 |
|  | Scg2 | | secretogranin II (chromogranin C) |
|  | Slc30a7 | | solute carrier family 30 (zinc transporter), member 7 |
|  | Soat1 | | sterol O-acyltransferase 1 |
|  | Trp53inp2 | | tumor protein p53 inducible nuclear protein 2 |
|  | Wdr44 | | WD repeat domain 44 |
|  | Wee1 | | wee 1 homolog (S. pombe) |
| miR-376a | Anxa6 | | annexin A6 |
|  | Decr1 | | 2,4-dienoyl CoA reductase 1, mitochondrial |
|  | Ephx1 | | epoxide hydrolase 1, microsomal |
|  | Mga | | MAX gene associated |
|  | Pon2 | | paraoxonase 2 |
|  | Nsdhl | | NAD(P) dependent steroid dehydrogenase-like |
|  | Csda | | cold shock domain protein A |
|  | Anxa2 | | annexin A2 |
|  | Plscr1 | | phospholipid scramblase 1 |
|  | LOC312273 | | Trypsin V-A |
|  | Notch1 | | Notch homolog 1, translocation-associated (Drosophila) |
|  | Gcg | | glucagon |
|  | Hmgcs2 | | 3-hydroxy-3-methylglutaryl-Coenzyme A synthase 2 (mitochondrial) |
|  | Ddt | | D-dopachrome tautomerase |
|  | Calr | | calreticulin |
|  | Tmem77 | | transmembrane protein 77 |
|  | Nfil3 | | nuclear factor, interleukin 3 regulated |
|  | Ssr1 | | signal sequence receptor, alpha |
|  | Idi1 | | isopentenyl-diphosphate delta isomerase 1 |
|  | Rbm17 | | RNA binding motif protein 17 |
|  | Rexo2 | | REX2, RNA exonuclease 2 homolog (S. cerevisiae) |
|  | Acaca | | acetyl-coenzyme A carboxylase alpha |
|  | Xpnpep1 | | X-prolyl aminopeptidase (aminopeptidase P) 1, soluble |
| miR-141 + miR-376a | Aldoa | | aldolase A, fructose-bisphosphate |
|  | Scg2 | | secretogranin II (chromogranin C) |
|  | Atp2c1 | | ATPase, Ca++ transporting, type 2C, member 1 |
| **Cluster IV** | **Gene** | | **Description** |
| miR-451 | Arih1 | | ariadne ubiquitin-conjugating enzyme E2 binding protein homolog 1 (Drosophila) |
|  | Copz2 | | coatomer protein complex, subunit zeta 2 |
|  | Cox17 | | cytochrome c oxidase, subunit XVII assembly protein homolog (S. cerevisiae) |
|  | Ctnnb1 | | catenin (cadherin associated protein), beta 1 |
|  | Cyb561 | | cytochrome b-561 |
|  | Dbt | | D site of albumin promoter (albumin D-box) binding protein |
|  | Deaf1 | | deformed epidermal autoregulatory factor 1 (Drosophila) |
|  | Gas6 | | growth arrest specific 6 |
|  | Hmgcs1 | | 3-hydroxy-3-methylglutaryl-Coenzyme A synthase 1 (soluble) |
|  | Il1rap | | interleukin 1 receptor accessory protein |
|  | Kctd3 | | potassium channel tetramerisation domain containing 3 |
|  | Lars | | leucyl-tRNA synthetase |
|  | Pgrmc1 | | progesterone receptor membrane component 1 |
|  | Pygl | | phosphorylase, glycogen, liver |
|  | Tacstd1 | | tumor-associated calcium signal transducer 1 |
|  | Tjp2 | | tight junction protein 2 |
|  | Hdlbp | | high density lipoprotein binding protein (vigilin) |
|  | Serpina6 | | serine (or cysteine) peptidase inhibitor, clade A, member 6 |
|  | Cand1 | | cullin-associated and neddylation-dissociated 1 |
|  | Hmgcs2 | | 3-hydroxy-3-methylglutaryl-Coenzyme A synthase 2 (mitochondrial) |
|  | Gbe1 | | glucan (1,4-alpha-), branching enzyme 1 |
|  | Hmgb2 | | high mobility group box 2 |
|  | Wif1 | | Wnt inhibitory factor 1 |
|  | Wdr44 | | WD repeat domain 44 |
|  | Idi1 | | isopentenyl-diphosphate delta isomerase 1 |
|  | Aass | | aminoadipate-semialdehyde synthase |
| miR-376b-3p | Cept1 | | choline/ethanolamine phosphotransferase 1 |
|  | Crot | | carnitine O-octanoyltransferase |
|  | Nfs1 | | NFS1 nitrogen fixation 1 homolog (S. cerevisiae) |
|  | Nucb2 | | nucleobindin 2 |
|  | Nsdhl | | NAD(P) dependent steroid dehydrogenase-like |
|  | Anxa2 | | annexin A2 |
|  | Plscr1 | | phospholipid scramblase 1 |
|  | LOC312273 | | Trypsin V-A |
|  | Ddt | | D-dopachrome tautomerase |
|  | Calr | | calreticulin |
|  | Nfil3 | | nuclear factor, interleukin 3 regulated |
|  | Xpnpep1 | | X-prolyl aminopeptidase (aminopeptidase P) 1, soluble |
|  | Ssr1 | | signal sequence receptor, alpha |
|  | Scg2 | | secretogranin II (chromogranin C) |
|  | Atp2c1 | | ATPase, Ca++ transporting, type 2C, member 1 |
|  | Notch1 | | Notch homolog 1, translocation-associated (Drosophila) |
|  | Rbm17 | | RNA binding motif protein 17 |
|  | Acaca | | acetyl-coenzyme A carboxylase alpha |
|  | Smpdl3a | | sphingomyelin phosphodiesterase, acid-like 3A |
|  | Tmem77 | | transmembrane protein 77 |
| miR-451 + miR-376b-3p | Gcg | | glucagon |

Genes common to multiple miRNAs as predicted by MiRanda and regulated in the perinatal pancreas.

| **Cluster I and II** |  |  |
| --- | --- | --- |
| **miRNA** | **Gene** | **Description** |
| miR-21, + miR-125b-5p | Plcb1  Hdc  Cyhr1 | phospholipase C, beta 1 (phosphoinositide-specific),  histidine decarboxylase  cysteine and histidine rich 1 |
| **Cluster I and III** |  |  |
| **miRNA** | **Gene** | **Description** |
| miR-29a + miR-141 | Cmas | cytidine monophosphate N-acetylneuraminic acid synthetase |
| miR-29a +miR-376a | Csda | cold shock domain protein A |
| miR-21 + miR-141 | Gcs1 | glucosidase 1 |
| miR-21 + miR-376a | Rexo2 | REX2, RNA exonuclease 2 homolog (S. cerevisiae) |
| **Cluster II and III** |  |  |
| **miRNA** | **Gene** | **Description** |
| miR-125b-5p + miR-141 | Soat1 | sterol O-acyltransferase 1 |
| miR-23a +miR-141 | Cryl1 | crystallin, lambda 1 |
| miR-23a + miR-141 | Rg9mtd2  Rnf138 | RNA (guanine-9-) methyltransferase domain containing 2  ring finger protein 138 |
| **Cluster II and IV** |  |  |
| **miRNA** | **Gene** | **Description** |
| miR-451 + miR-125b-5p | Hdlbp  Serpina6 | high density lipoprotein binding protein (vigilin)  serine (or cysteine) peptidase inhibitor, clade A, member 6 |
| miR-451 + miR-23a | Cand1  Hmgb2 | cullin-associated and neddylation-dissociated 1  high mobility group box 2 |
| **Cluster III and IV** |  |  |
| **miRNA** | **Gene** | **Description** |
| miR-376b-3p + miR-141+ miR-376a | Scg2  Atp2c1 | secretogranin II (chromogranin C)  ATPase, Ca++ transporting, type 2C, member 1 |
| miR-451, +miR-141 | Wdr44 | WD repeat domain 44 |
| miR-376b-3p + miR-376a | Nsdhl  Anxa2  Plscr1  LOC312273  Notch1  Ddt  Calr  Nfil3  Ssr1  Rbm17  Acaca  Xpnpep1 | NAD(P) dependent steroid dehydrogenase-like  annexin A2  phospholipid scramblase 1  Trypsin V-A  Notch homolog 1, translocation-associated (Drosophila)  D-dopachrome tautomerase  Calreticulin  nuclear factor, interleukin 3 regulated  signal sequence receptor, alpha  RNA binding motif protein 17  acetyl-coenzyme A carboxylase alpha  X-prolyl aminopeptidase (aminopeptidase P) 1, soluble |
| miR-451 + miR-376b-3p + miR-376a | Gcg | Glucagon |
| miR-451 + miR-376a | Idi1 | isopentenyl-diphosphate delta isomerase 1 |
| **Cluster I and IV** |  |  |
| **miRNA** | **Gene** | **Description** |
| miR-29a + miR-376b-3p | Smpdl3a | sphingomyelin phosphodiesterase, acid-like 3A |
| **Cluster I, III and IV** |  |  |
| **miRNA** | **Gene** | **Description** |
| miR-21 + miR-451 +  miR-141 | Gbe1 | glucan (1,4-alpha-), branching enzyme 1 |
| miR-21 + miR-451 +  miR-376a | Hmgcs2 | 3-hydroxy-3-methylglutaryl-Coenzyme A synthase 2 (mitochondrial) |
| **Cluster I, II, III and IV** |  |  |
| **miRNA** | **Gene** | **Description** |
| miR-21 + miR-376b-3p + miR-125b-5p +  miR-376a | Tmem77 | transmembrane protein 77 |
